# Micronutrient Status in Patients with Severe Obesity Before and After Laparoscopic Sleeve Gastrectomy

**DOI:** 10.3390/nu16244386

**Published:** 2024-12-20

**Authors:** Daniela Ciobârcă, Adriana Florinela Cătoi, Cătălin Copăescu, Mihaela Iancu, Ioana Delia Pop, Dan Cristian Vodnar, Andra Diana Cecan, Doina Miere, Lorena Filip, Gianina Crișan

**Affiliations:** 1 Department 2, Faculty of Nursing and Health Sciences, “Iuliu Hatieganu” University of Medicine and Pharmacy, 400337 Cluj-Napoca, Romania; muresan.daniela@umfcluj.ro; 2 Department of Pathophysiology, Faculty of Medicine, “Iuliu Hatieganu” University of Medicine and Pharmacy, 400012 Cluj-Napoca, Romania; andra.cecan@umfcluj.ro; 3 Center of Excellence in Bariatric and Metabolic Surgery, Ponderas Academic Hospital, 014142 Bucharest, Romania; president@rsms.ro; 4 Medical Informatics and Biostatistics, Department 1, Faculty of Nursing and Health Sciences, “Iuliu Hațieganu” University of Medicine and Pharmacy, 400349 Cluj-Napoca, Romania; 5 Department of Exact Sciences, University of Agricultural Sciences and Veterinary Medicine, 400372 Cluj-Napoca, Romania; popioana@usamvcluj.ro; 6 Department of Food Science, Faculty of Food Science and Technology, University of Agricultural Sciences and Veterinary Medicine, 400372 Cluj-Napoca, Romania; dan.vodnar@usamvcluj.ro; 7 Departament of Bromatology, Hygiene, Nutrition, Faculty of Pharmacy, “Iuliu Hatieganu” University of Medicine and Pharmacy, 400337 Cluj-Napoca, Romania; dmiere@umfcluj.ro (D.M.); lfilip@umfcluj.ro (L.F.); 8 Department of Pharmaceutical Botany, Faculty of Pharmacy, “Iuliu Hatieganu” University of Medicine and Pharmacy, 400337 Cluj-Napoca, Romania; gcrisan@umfcluj.ro

**Keywords:** micronutrient deficiencies, laparoscopic sleeve gastrectomy, metabolic bariatric surgery, obesity

## Abstract

**Background:** Micronutrient deficiencies (MNDs) are commonly reported after bariatric and metabolic surgery, including laparoscopic sleeve gastrectomy (LSG). Nevertheless, the micronutrient status changes over time and the influence of sex or initial body mass index (BMI) on these changes are less explored. This study aims to investigate the changes in micronutrient levels at 6 and 12 months after LSG and the potential influence of sex or baseline BMI (≥40 kg/m^2^) on these changes in patients submitted to LSG. Additionally, the frequency of MNDs before and at 12 months after the procedure was investigated. **Materials and methods**: Fifty patients with obesity underwent LSG and were assessed anthropometrically and nutritionally at baseline and at 6 and 12 months, respectively, after LSG. The changes in micronutrients levels over time were tested by a linear mixed model. **Results**: Vitamin B_12_ and vitamin D [25(OH)D] did not change significantly, while iron (*p* < 0.001), calcium (*p* = 0.01), and parathormone (*p* < 0.001) differed significantly from baseline to 12 months after LSG. Ferritin significantly decreased from baseline to 6 months and 12 months after LSG (LS-means, 95% CI: 202 [163, 240] vs. 160 [130, 191] vs. 150 [115, 185]). Sex or initial severe obesity (BMI ≥ 40 kg/m^2^) exhibited significant modifying effects for 25(OH)D and calcium, respectively. The 25(OH)D levels increased significantly in men, but not in women, while the calcium plasma concentration changed significantly only in patients with initial severe obesity. No significant changes over time were found for MNDs’ frequency (*p* > 0.05). The most consistent deficiency frequency was observed for 25(OH)D both before and after LSG. **Conclusions**: Overall, our findings revealed changes in micronutrient status across the follow-up period, except for vitamin B_12_. Variations in 25(OH)D levels were reported exclusively in men, suggesting that they depend on sex. The calcium plasma concentration showed significant changes exclusively in patients with BMI ≥ 40 kg/m^2^. MNDs’ frequency was not significantly altered during the study follow-up. Our results reinforce the need for developing national dietary guidelines tailored for Romanian patients following LSG.

## 1. Introduction

Obesity, one of the most serious public health issues globally, strongly contributes to the development of cardiovascular disease, type 2 diabetes (T2D), and some cancers, which can cause substantial disability and premature death [1]. Since 1975, prevalence estimates for overweight and obesity have risen by 51% in the European region, with almost 60% of adults facing challenges related to excess body weight [2]. In Romania, 34.7% of the adult population is living with obesity [3].

Metabolic and bariatric surgery (MBS) has emerged as a highly effective treatment for severe obesity and is indicated in patients with a body mass index (BMI) ≥ 35 kg/m^2^, irrespective of the presence or severity of associated comorbidities. MBS is also recommended in patients with T2D and BMI ≥ 30 kg/m^2^ and should be taken into account in patients with obesity class I (BMI ≥ 30 kg/m^2^) without significant or long-lasting weight loss and comorbidity improvements following non-surgical interventions [4]. Laparoscopic sleeve gastrectomy (LSG) reduces the gastric capacity by 80% [5] but also acts via complex neuro-hormonal mechanisms, demonstrating similar benefits in terms of weight loss and improvements in metabolic-related complications to those of more invasive interventions [6]. Furthermore, LSG is linked to a lower rate of complications than malabsorptive treatments [7] and is currently the most performed MBS procedure in Romania as well as worldwide [8].

Micronutrient deficiencies (MNDs) are commonly reported both before and after MBS [9,10,11,12]. Patients with severe obesity undergoing MBS exhibit greater incidences of MNDs than individuals with normal weight due to several factors, including poor food habits, increased micronutrient demands, impaired micronutrient metabolism, and low-grade inflammation [13]. The prevalence of MNDs prior to MBS is reported to be high [14,15], with vitamin D [25(OH)D], iron, folate, and vitamin B_12_ being the major MNDs described in these patients [9,16,17]. MNDs are also frequently reported following bariatric interventions, including LSG [13,18,19]. The incidences of MNDs after the latter procedure can exceed 70% for 25(OH)D [20], 50% for anemia predominantly induced by iron, vitamin B_12_, and vitamin B_9_ deficiencies [21], or 18% for calcium [22]. Nevertheless, it has been hypothesized that the likelihood of acquiring MNDs after LSG is lower than that after malabsorptive procedures [23]. In fact, compared to laparoscopic Roux-en-Y gastric bypass (LRYGB), LSG has a decreased postoperative nutritional risk according to several studies [24]. Nevertheless, because of differences in study design (e.g., type of nutrients evaluated, the length of the follow-up, or the dietary supplements prescribed following surgery), there is a large degree of heterogeneity in the present literature in terms of the micronutrient status after LSG [18]. Some authors argue that long-term supplementation is required after LSG, while others contend that LSG does not significantly alter the micronutrient status and that, as a result, nutritional supplementation should not be continued for longer than three months postoperatively [25] However, since all bariatric procedures alter to a variable extent the anatomy and physiology of the gastrointestinal tract, MNDs are commonly reported in individuals submitted to these interventions [19].

According to clinical findings, patients undergoing LSG experience deficiencies in iron and folate as well as in vitamin B_12_ [23,26,27]. Low serum ferritin, which measures how much iron is stored, is a direct sign of iron deficiency [28]. Notably, one of the most common side effects of BS is anemia reflected by low hemoglobin (Hb) levels [29]. Vitamin D deficiency associated with hypocalcemia and increased parathormone (PTH) levels is another significant deficiency that has been documented both before and after LSG [26]. Furthermore, the preoperative values of each parameter are independently correlated with the majority of MNDs following LSG [30]. Therefore, if the preoperative MNDs are not identified and treated accordingly, they may worsen after MBS [12,18]. As stated by the current guidelines, vitamin and mineral supplementation is required as LSG is linked to the risk of developing MNDs [31,32]. Nevertheless, patients undergoing LSG may experience MNDs even with supplementation [18]. On the other hand, an ideal supplement to meet the nutritional needs of all LSG patients does not exist [33]. Poor compliance to supplementation, which has been observed to be as low as 20% postoperatively, may also be responsible for MNDs’ development [34].

The objectives of this study were to determine if the micronutrient levels in patients with obesity change before and at 6 and 12 months following LSG and whether this change can be influenced by sex or initial BMI (defined as BMI ≥ 40 kg/m^2^ vs. BMI 35–40 kg/m^2^). Also, we investigated if the MNDs’ frequency differs before and at 12 months after LSG.

## 2. Materials and Methods

This observational longitudinal study analyzed retrospectively the prospective database of the patients with severe obesity who underwent LSG at the Center of Excellence in Bariatric and Metabolic Surgery Ponderas Academic Hospital Bucharest (Romania). Fifty patients fulfilling the BMI-based criteria for BS endorsed by the International Federation for the Surgery of Obesity (IFSO) [35] who underwent LSG between 2014 and 2015 using the same technique as previously described [36] and agreeing to the study’s protocol were included in this study.

The patients were evaluated preoperatively and at six and 12 months after LSG. Assessments performed at pre-surgery as well as post-surgery appointments included body weight, height, waist and hip circumference measurements, blood sample collection, and nutritional evaluation. Demographic, anthropometric, and micronutrient status data were collected from medical electronic records. Each patient provided informed consent to participate in this study. All procedures were carried out in compliance with the ethical standards of Ponderas Academic Hospital and with the Declaration of Helsinki principles. The Ethics Committee of Ponderas Academic Hospital (258/20.12.2013) and of “Iuliu Hatieganu” University of Medicine and Pharmacy Cluj-Napoca (Romania) granted approval for this study (186/19.04.2018).

### 2.1. Anthropometric Indices

BMI as well as accepted parameters to assess postoperative weight loss [excess weight loss (%EWL), excess BMI loss (%EBMIL), and total weight loss (%TWL)] were calculated as follows:

BMI = weight (kg)/height (m)^2^

%EWL = [(initial weight − postop weight)]/(initial weight − ideal weight)] × 100

%EBMIL = [(initial BMI − postop BMI)]/(initial BMI − 25)] × 100

%TWL = (weight loss/the initial weight) × 100

The weight corresponding to a BMI of 25 kg/m^2^ was considered ideal [37,38].

Waist and hip circumferences were measured at the umbilical and the greater trochanter level using a flexible measuring tape. Two repeated measurements were performed at each site level. The waist to hip ratio (WHR) was calculated as follows:

WHR = waist measurement (cm)/hip measurement (cm)

A WHR above 0.83 in females and 0.9 in males is considered a strong predictor of cardiovascular events [37].

### 2.2. Laboratory Measurements

Blood samples were centrifuged and run immediately or otherwise stored at −80 °C until the corresponding tests were performed. The biochemical assays consisted of complete blood count, Hb, iron, ferritin, serum folate, vitamin B_12_, calcium, 25(OH)D, and PTH. Laboratory measurements were conducted at the Ponderas Academic Hospital laboratory and at the Biogen Medical Laboratory (Cluj-Napoca, Romania). Deficiency or excess in a micronutrient or hormone was considered if the serum level of the respective micronutrient was below or above the laboratory reference range (Table 1).

The primary study outcomes were defined as follows:

- iron deficiency: diagnosed based on iron levels below 50 μg/dL as well as on ferritin levels with threshold <28 ng/mL (male) and <5 ng/mL (female);
- anemia: defined according to the WHO criteria of Hb levels < 12 g/dL in women and <13 g/dL in men, respectively [39];
- 25(OH)D status: characterized as deficient if below <10 ng/mL, insufficient if between 10 and 29 ng/mL, and optimal if >30 ng/mL;
- secondary hyperparathyroidism: diagnosed based on excess PTH levels (>64 pg/mL) in the presence of low calcium (<8.6 mg/dL) or 25(OH)D (<30 ng/mL).

### 2.3. Nutritional Evaluation and Supplementation

An interdisciplinary team conducted a nutritional assessment on all patients at baseline and during follow-up appointments. Dietary recommendations concerning food choices, hydration, and micronutrient deficiency prevention were given at each visit. The standard postoperative supplementation protocol included one multivitamin and mineral tablet daily during the first year following LSG as well as daily oral calcium citrate (500 mg), iron (28 mg), vitamin D (2000 IU), and vitamin B_12_ (1000 μg) along the first three months postoperatively. The results of the biochemical testing were used to match dietary supplementation to each patient’s specific nutritional needs throughout the follow-up phase. Extra micronutrients were added to the initial supplementation regimen in certain instances.

### 2.4. Statistical Analysis

Arithmetic or geometric means were used as centrality measures for quantitative continuous variables with normal or log-normal distributions. Median with interquartile range defined as [25th percentile, 75th percentile] was used to summarize data with deviations from the normal distribution. The fit of empirical data distribution with univariate normal distribution or log-normal distribution was tested using descriptive statistics, a quantile–quantile (Q-Q) plot, skewness–kurtosis plot, Shapiro–Wilk’s test, and the computation of different goodness-of-fit statistics to evaluate the distance between the fitted parametric distribution and the empirical data distribution. Qualitative variables were summarized by absolute frequencies and percentages (%).

To test the differences in the anthropometric and nutritional parameters over time (at months and at 12 months after LSG), we used the linear mixed models (LMMs) and generalized linear mixed models (GLMMs) with repeated-measures design on time and patient-level random intercept. Time points (defined as baseline and at six months and 12 months), sex (male vs. female), and baseline BMI (analyzed as a dichotomous variable with the following categories: BMI ≥ 40 kg/m^2^ vs. BMI 35–40 kg/m^2^) were considered as the main effects in all the tested linear mixed models. We also tested the interaction effect of time with sex and baseline BMI to estimate how changes in the micronutrient profile depend on the sex or baseline BMI group. Pairwise comparisons using least-square means (LSMs) with Tukey’s HSD correction were tested for differences within each group over time and between groups at each time point using the lme4 package (version 1.1-35.5) [40] and emmeans package (version 1.8.8) [41] in R software. The results were quantified using the least-square means with a 95% confidence level (95% CI). Missing data were supposed to be missing at random, and pairwise deletion was applied in the GLMM.

Significant changes in the proportions of MNDs between the baseline and at 12 months after LSG were identified by a pairwise McNemar test.

All the results of the two-sided statistical tests were interpreted with a 5% level of significance (α) and a 95% level of confidence. Statistical analyses were performed using R statistical software (R Foundation for Statistical Computing, Vienna, Austria, version 4.2.3).

## 3. Results

### 3.1. Sample Characteristics

In the present study, the mean age of the patients was 46.5 years (SD = 10.9). Patients’ ages ranged from 22 to 66 years old. Out of the total 50 patients, 34 (68%) were females and the remaining 16 (32%) were males. The baseline measurements showed a mean BMI of 43.4 kg/m^2^ (SD = 5.6) (min–max: 34.5–57.3 kg/m^2^) and a mean WHR of 1.1 (0.1) for men and 0.9 (0.1) for women (Table 2). There were 36 patients (72%) with severe obesity and 14 (28%) with obesity class II. Also, 12 patients (24%) displayed T2D [out of which 6 cases (50%) were under medication], 25 (50%) hypertension [out of which 20 cases (80%) with medication], and 44 (88%) dyslipidemia [out of which 12 cases (27%) with medication] (Table 2).

### 3.2. Longitudinal Change in Anthropometric Characteristics in All Obese Patients’ Samples

The linear mixed models revealed a main significant association of time with weight and BMI (*p* < 0.0001 at 12 months and six months after LSG vs. baseline). A significant change in WHR was noticed only from baseline to months after LSG (Table 3). Post hoc pairwise comparisons revealed a significant gradual reduction in mean BMI from baseline to six months after LSG [mean difference = −12.3 kg/m^2^, 95% CI: −13.1 to −14.4] and from baseline to 12 months after LSG [mean difference: −15.3 kg/m^2^, 95% CI: −16.5 to −14.1] in all samples. Likewise, we found a significant main time effect (*p* < 0.001) on the evolution of %EWL, %EBMIL, and %TWL at 12 months vs. six months after LSG.

### 3.3. Longitudinal Change in Anthropometric Characteristics Stratified by Sex

The comparison according to sex group (males vs. females) at each time point showed significant differences between males and females concerning weight (Table 3) and WHR (*p* < 0.001) and no significant difference regarding %EWL (*p* = 0.53), %EBMIL (*p* = 0.56), and %TWL (*p* = 0.55). The LS-mean of weight was significantly lower in women compared to men (83.1, 95% CI: [79.1, 87.4] kg vs. 98.5, 95% CI: [91.8, 106.7] kg and 73.7, 95% CI: [70.1, 78.3] kg vs. 91.8, 95% CI: [85.6, 99.5] kg) at six months and at 12 months after LSG.

**Figure 1:**
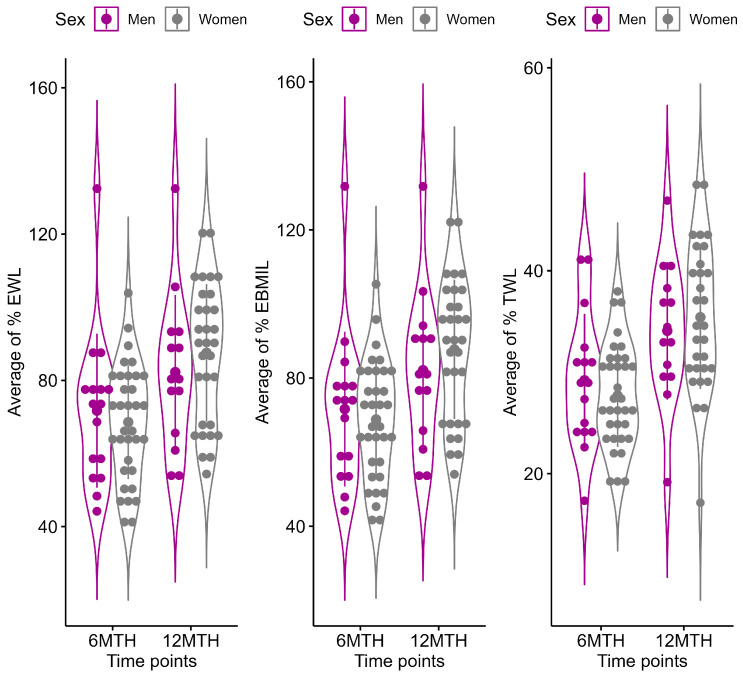
Changes in %EWL, %EBMIL, and %TWL during the post-surgery period. Results are represented as violin plots. Solid error bars on plots represent mean value (observed mean) with standard deviation calculated at each time points (6 MTH = at six months and 12 MTH = at 12 months after LSG) for males (magenta color) and females (grey color).

The LMM indicated a significant time by sex interaction effect for %EWL (*p* interaction = 0.003), %EBMIL (*p* interaction = 0.003), and %TWL (*p* interaction = 0.009) (Supplementary Table S1). A significant change from six months to 12 months after LSG was found to be higher in females than in males for %EWL (male group: LS-means difference = −9.7%, adjusted *p* = 0.001 vs. female group: LS-means difference = −19.1%, adjusted *p* < 0.001), %EBMIL (male group: LS-means difference = −9.7%, adjusted *p* = 0.001 vs. female group: LS-means difference = −18.97%, adjusted *p* < 0.001), and %TWL (male group: LS-means difference = −4.32%, adjusted *p* = 0.002 vs. female group: LS-means difference = −7.9%, adjusted *p* < 0.001) (Figure 1).

### 3.4. Changes in Hematinic and Hematological Values over Time in All Patients’ Samples

The results of the main effects analysis indicated that there were significant changes over time for folate (*p* = 0.012), iron (*p* = 0.002), ferritin (*p* = 0.04), MCV (*p* = 0.02), and MCH (*p* = 0.045). Post hoc comparisons performed by Tukey’s test showed that the mean folate value increased from the baseline to six months after LSG (LS-means, 95% CI: 5.8 [4.3, 7.9] vs. 8.7 [6.8, 11.2], adjusted *p* < 0.001) and decreased significantly from six months to 12 months after LSG (LS-means, 95% CI: 8.7 [6.8, 11.2] vs. 5.8 [4.2, 8.1], adjusted *p* = 0.001). There was a significant difference in the iron mean values from the baseline to 12 months after LSG (LS-means, 95% CI: 88.6 [77.9, 99.2] vs. 113.8 [103.0, 124.6], adjusted *p* < 0.001) and from six months to 12 months after LSG (LS-means, 95% CI: 98.6 [88.1, 109.1] vs. 113.8 [103.0, 124.6], adjusted *p* = 0.04), whereas the change from the baseline to six months was not significant (adjusted *p* = 0.25). Ferritin decreased from the baseline to six months and 12 months after LSG (LS-means, 95% CI: 202 [163, 240] vs. 160 [130, 191] vs. 150 [115,185]). Regarding MCV changes over time, a significant difference was found only from the baseline to six months after LSG (LS-means, 95% CI: 89.3 [88.0, 90.6] vs. 90.9 [89.5, 92.2]).

### 3.5. Longitudinal Changes in Hematinic and Hematological Values by Sex Group and Baseline BMI

The comparisons by sex (males vs. females) and by initial BMI (≥40 kg/m^2^ vs. 35–40 kg/m^2^) at each time point (baseline and at six and 12 months) are described in Table 4. There was a significant interaction effect between time and initial BMI on hematocrit (Hct) levels (*p* interaction = 0.046). The mean difference in Hct from the baseline to six months (adjusted *p* = 0.002) and from the baseline to 12 months (adjusted *p* < 0.001) was significant only for obese patients with an initial BMI ≥ 40 kg/m^2^ (Supplementary Table S1). There was no significant interaction effect between time and sex or time and initial BMI on vitamin B_12_, folate, iron, ferritin, Hb, MCV, and MCH levels (*p* interaction > 0.05, Supplementary Table S2).

### 3.6. Longitudinal Changes in Vitamin D–Calcium Status and PTH Values in All Patients’ Samples

Significant changes over time were found for PTH and calcium levels (*p* < 0.05). Post hoc analysis performed by Tukey’s test indicated a significant increase in the mean values of PTH from the baseline to 6 months and to 12 months after LSG (LS-means, 95% CI: 27.8 [22.8, 32.8] vs. 34.5 [29.6, 39.4] vs. 40.0 [34.7, 45.3], adjusted *p* = 0.04 and adjusted *p* < 0.001, respectively). Similar results were also found for calcium from the baseline to six months (LS-means, 95% CI: 9.1 [8.9, 9.2] vs. 9.4 [9.3, 9.6] vs. 9.3 [9.2, 9.5], adjusted *p* < 0.001) and from the baseline to 12 months after LSG (LS-means, 95% CI: 9.1 [8.9, 9.2] vs. 9.3 [9.2, 9.5], adjusted *p* = 0.01).

### 3.7. Longitudinal Changes in Vitamin D–Calcium Status and PTH Values by Sex and Baseline BMI

The comparisons by sex (males vs. females) and by initial BMI (≥40 kg/m^2^ vs. 35–40 kg/m^2^) concerning 25(OH)D, PTH, and calcium at each time point (baseline and at six and 12 months) were described in Table 4. The effect of time on the 25(OH)D levels varied significantly depending on sex (*p* interaction < 0.001) (Supplementary Table S2). The changes in vitamin 25(OH)D over time were significantly different according to sex, i.e., the mean difference from the baseline to 12 months (adjusted *p* < 0.001) and from six months to 12 months (adjusted *p* < 0.001) was significant only for men (not for women, Table 4). We found no significant interaction effect between time and initial BMI on the 25(OH)D levels (*p* interaction > 0.05, Supplementary Table S2). With respect to calcium, a significant interaction between initial BMI and time was found (*p* interaction = 0.02), i.e., there was a significant change from the baseline to six months (adjusted *p* < 0.001) and from the baseline to 12 months (adjusted *p* < 0.001), but only for obese patients with initial BMI ≥ 40 kg/m^2^ (Table 4). We found no significant interaction effect between time and sex or between time and initial BMI on PTH levels (*p* > 0.05, Supplementary Table S2).

**Table 4:** Changes in 25(OH)D, PTH, and calcium after LSG stratified by sex and initial BMI level.

Outcome	Subgroup	Baseline	6 MTH	*p*-Value ^1^	12 MTH	*p*-Value ^2^	*p*-Value ^3^	Adjusted *p*-Values (Adjusted for Sex and BMI ≥ 40 kg/m^2^)		
		LS-Means [95% CI]	LS-Means [95% CI]		LS-Means [95% CI]			Baseline	6 MTH	12 MTH
25(OH)D (ng/mL) ^a^	Male	8.8 [5.0, 15.3] ^#^	11.2 [7.9, 16.0] ^#^	0.76	20.6 [14.7, 28.7] ^#^	<0.001 *	<0.001 *	0.99	0.92	0.001 *
	Female	9.9 [6.5, 4.9] ^#^	9.9 [7.5, 13.0] ^#^	1.00	10.6 [8.1, 13.8] ^#^	0.98	>0.99			
	BMI ≥ 40 kg/m^2^	8.8 [4.6, 17.0] ^#^	11.4 [9.0, 14.3] ^#^	0.77	14.3 [11.3, 18.1] ^#^	0.08	0.03 *	>0.99	0.90	>0.99
	BMI 35–40 kg/m^2^	9.8 [6.9, 13.8] ^#^	9.8 [6.5, 14.8] ^#^	>0.99	15.3 [10.5, 22.1] ^#^	0.03 *	0.14			
PTH (pg/mL) ^b^	Male	29.8 [22.1, 37.5]	34.5 [27.2, 42.5]	0.84	41.2 [32.6, 49.7]	0.76	0.15	0.95	1.00	>0.99
	Female	25.8 [20.3, 31.2]	34.1 [28.8, 39.5]	0.07	38.8 [33.3, 44.4]	0.64	<0.001 *			
	BMI ≥ 40 kg/m^2^	30.3 [25.2, 35.4]	34.1 [29.0, 39.2]	0.75	40.1 [34.5, 45.7]	0.38	0.02 *	0.90	1.00	1.00
	BMI 35–40 kg/m^2^	25.3 [17.0, 33.5]	34.9 [26.8, 43.0]	0.29	39.9 [31.4, 48.4]	0.89	0.03 *			
Calcium (mg/dL) ^c^	Male	9.4 [9.2, 9.6]	9.4 [9.2, 9.6]	0.046 *	9.4 [9.2, 9.6]	1.00	0.09	0.99	>0.99	0.86
	Female	9.1 [8.9, 9.3]	9.5 [9.3, 9.6]	0.004 *	9.3 [9.1, 9.4]	0.22	0.65			
	BMI ≥ 40 kg/m^2^	9.0 [8.9, 9.1]	9.4 [9.3, 9.5]	<0.001 *	9.4 [9.3, 9.6]	>0.99	<0.001 *	0.79	>0.99	0.59
	BMI 35–40 kg/m^2^	9.2 [8.9, 9.4]	9.5 [9.2, 9.7]	0.23	9.2 [9.0, 9.5]	0.49	>0.99			

*n* = number of patients; *n*_1_ = number of patients in the first subgroup; *n*_2_ = number of patients in the second subgroup; 6 MTH = at six months and 12 MTH = at 12 months after LSG; ^1^ adjusted *p*-values for change from baseline to 6 MTH; ^2^ adjusted *p*-values for change from 6 MTH to 12 MTH; ^3^ adjusted *p*-values for change from baseline to 12 MTH. * significant result: *p* < 0.05; ^a^ T0: *n* = 24 (sex: *n*_1_ = 9, *n*_2_ = 15; BMI: *n*_1_ = 18, *n*_2_ = 6), T6: *n* = 49 (sex: *n*_1_ = 15, *n*_2_ = 34; BMI: *n*_1_ = 35, *n*_2_ = 14), T12: *n* = 39 (sex: *n*_1_ = 9, *n*_2_ = 30; BMI: *n*_1_ = 27, *n*_2_ = 12); ^b^ T0: *n* = 49 (sex: *n*_1_ = 16, *n*_2_ = 33; BMI: *n*_1_ = 36, *n*_2_ = 13), T6: *n* = 50 (sex: *n*_1_ = 16, *n*_2_ = 34; BMI: *n*_1_ = 36, *n*_2_ = 14), T12: *n* = 42 (sex: *n*_1_ = 12, *n*_2_ = 30; BMI: *n*_1_ = 29, *n*_2_ = 13); ^c^ T0: *n* = 48 (sex: *n*_1_ = 16, *n*_2_ = 32; BMI: *n*_1_ = 35, *n*_2_ = 13), T6: *n* = 49 (sex: *n*_1_ = 16, *n*_2_ = 33; BMI: *n*_1_ = 35, *n*_2_ = 14), T12: *n* = 43 (sex: *n*_1_ = 12, *n*_2_ = 31; BMI: *n*_1_ = 30, *n*_2_ = 13); ^#^ estimated least-square means (LS-means) for log-normal data are given on the response scale (after we exponentiated the results obtained for log scale); *p*-values at each time point were estimated using Tukey’s HSD correction.

### 3.8. Differences in MNDs over Time

The frequencies of MNDs were not significantly different between the baseline and 12 months after LSG, as shown in Table 5. In terms of hematological parameters, namely Hb and Hct, no significant changes in preoperative and postoperative deficiency were identified [Hb two cases (4.3%) vs. four cases (8.7%), *p* = 0.62, and Hct two cases (4.3%) vs. six cases (13.0%), *p* = 0.13]. Excess PTH was observed in one case (2.4%) at the baseline and at 12 months after LSG, respectively, (*p* > 0.05).

## 4. Discussion

Herein, we investigated the hematinic status (vitamin B_12_, folate, iron, ferritin), 25(OH)D-calcium status, anemia markers, and PTH levels as well as the frequency of MNDs before and 12 months following LSG. Weight loss was significantly higher in females vs. males. The main findings of this study were as follows:

(i) Folate and vitamin B_12_ levels did not modify substantially between the baseline and 12 months after LSG; serum iron increased, and ferritin decreased, although in keeping with the normal range. The effect of time on the vitamin B_12_, folate, iron, and ferritin levels did not vary significantly across the different sexes or initial BMI categories. The significantly low levels of 25(OH)D documented prior to LSG did not suffer variation during the follow-up period. Although we did observe a significant increase in 25(OH)D from the baseline to the end of the follow-up period in men, its mean value remained low at 12 months following LSG. Throughout the course of this study, there was an increase in the mean calcium levels along with a significant increase in PTH, while remaining within the normal range.
(ii) The frequency of several MNDs, folate (6.6 vs. 11.1%), vitamin B_12_ (16.6 vs. 12.5%), iron (2.2 vs. 4.5%), calcium (4.6 vs. 2.3%), and 25(OH)D (100 vs. 100%), respectively, did not modify significantly from the baseline to 12 months after LSG. Ferritin deficiency was not observed. PTH excess was identified in only one case (2.4%) both before and after 12 months following LSG.

The most frequent MNDs after LSG include vitamin B_12_, folate, vitamin B_6_, 25(OH)D, and iron [42]. Several factors affect the micronutrient status of individuals after LSG, such as dramatically decreased food intake, vomiting, food intolerance, altered gastric secretion, or the rapid transit of recently ingested food within the gastrointestinal tract [30]. Moreover, the discontinuation of vitamin and mineral supplementation after LSG may also be responsible for the occurrence of MNDs. A recent meta-analysis highlighted that the rates of guideline adherence to micronutrient supplementation after BS are as low as 20% [34].

In our study, the relative frequencies of vitamin B_12_ deficiency were low both prior and after 12 months following LSG (16.6% vs. 12.5%), with no significant change throughout the study period. Previous research reported that vitamin B_12_ deficiency prevalence 12 months after LSG ranged between 0 and 26.2% [43]. In line with earlier studies [42,44], we observed that the mean serum levels of vitamin B_12_ were within the optimal range without significant variation across the follow-up period. Since vitamin B_12_ (cobalamin) plays an essential role in the enzymatic reactions required for red blood cell synthesis and myelination, its deficiency results in anemia from ineffective erythropoiesis and impaired neurological function (e.g., peripheral neuropathy) [45,46]. A decrease in vitamin B_12_ absorption capacity is one of the factors responsible for the onset of deficiency-related disturbances in bariatric patients [47]. Vitamin B_12_ is absorbed in the distal ileum, requiring the presence of the intrinsic factor (IF) released by the parietal cells of the stomach. Gastric fundus resection secondary to LSG impairs IF secretion and subsequent vitamin B_12_ absorption [12]. Not least, a decreased intake of animal protein because of poor intolerance could exacerbate vitamin B_12_ deficiency following LSG [47]. Nevertheless, the rather normal vitamin B_12_ status that we observed in our study can be accounted for by the hepatic and renal vitamin B_12_ storages and post-surgery nutritional supplementation. Indeed, following absorption, vitamin B_12_ is stored in large amounts in the liver such that anemia might not develop until after a couple of years of deficiency [48]. Both Damms-Machado et al. [42] and Gillon et al. [27] reported an increase in vitamin B_12_ deficiency percentage from the baseline to 12 months after LSG, from 9.3% to 17.2% and from 6.4% to 19%, respectively. However, consistent with our findings, a recent systematic review showed that most studies investigating vitamin B_12_ deficiency prevalence (9/10) reported no significant alteration of B_12_ status throughout the first 12 months after LSG [43]. Vitamin B_12_ deficiency is commonly reported to develop between 12 and 36 months following LSG, as measured by total plasma B_12_ [12,24,49]. Finally, some controversy also surrounds the effectiveness of routes for vitamin B_12_ supplementation. Hakeam et al. [50] observed that 26% of patients who underwent LSG developed vitamin B_12_ deficiency at 12 months, although cobalamin was orally supplemented five times the recommended daily allowance. According to a meta-analysis conducted by Kwon et al. [51], intramuscular B_12_ supplementation was more effective in correcting the deficiency than oral B_12_ (91% vs. 81% of cases).

The metabolic activities of vitamin B_12_ and folate (vitamin B9) are closely related at the cellular level as the former is responsible for activating folic acid by converting methyltetrahydrofolic acid into tetrahydrofolic acid [52,53]. The percentage of folate deficiency, similar to that of vitamin B_12_, was found to be relatively low at the end of the follow-up period. Compared to the baseline, the folate deficiency frequency increased from 6.6% to 11.1% at 12 months after LSG with no significant difference between the two moments. Similar findings were reported by Gillon et al. [27], with 8.8% at the baseline vs. 12.3% at 12 months following LSG, respectively. However, several other studies showed a higher proportion of patients with folate deficiency across the same follow-up period, with a prevalence ranging from 13.8% at the baseline to 21.4% at 12 months after LSG [18,30,44,54]. Most studies included in the meta-analysis conducted by Lewis et al. [43] also indicated that the serum folate levels did not vary significantly between the baseline and 1 months postoperatively. On the other hand, Dong et al. [44] found a significant increase in folate deficiency from the baseline to twelve months after LSG [7.7% to 19.2%], while van Rutte et al. [12] reported an opposite trend, with a significant decrease from 23.9% at the baseline to 12.4% at 12 months post-LSG. These inconsistencies could probably be explained by variations in food intake and postoperative supplementation. According to our findings, the mean folate serum levels increased significantly at the first time point (6 months) and then decreased by the end of the follow-up period; however, the mean folate values from the baseline to 12 months after LSG showed no change and remained within the normal reference range, in line with other studies [18,54]. There are a few possible explanations for the low folate deficiency documented in our study. Folate deficiency, which is expected to develop earlier after LSG than B_12_ deficiency [55], is primarily caused by inadequate dietary intake and not by the surgery itself [27]. Still, the mechanism of the intestinal absorption of dietary folate involves a pH-dependent carrier which may be affected by the reduced gastric hydrochloric acid synthesis secondary to LSG [56]. As mentioned earlier, since the metabolism of vitamin B_12_ is closely entwined with that of folic acid, the deficiency of one can impair the other [55]. Finally, low adherence to post-LSG supplementation is a possible explanation of the mean folate values trend observed in our study. The decrease in the mean folate values during the second half of the follow-up period could indicate that adherence to nutritional supplementation deteriorated over time.

Iron deficiency is not only one of the most frequently reported MNDs after LSG [23] but also the most common causative factor of anemia, along with vitamin B_12_ deficiency [57]. Studies investigating iron status in bariatric patients submitted to LSG reported a high prevalence of iron and ferritin deficiency, ranging from 44% and 28.2% at the baseline to 41% and 34% at 12 months postoperatively [26,43,58]. Analysis of serum iron and ferritin showed a low percentage of iron deficiency at the baseline and at the end of the follow-up period (2.2% and 4.5%, respectively) without a significant variation between the initial and final time point. This was consistent with the data reported by Dong et al. [44], which showed a deficiency prevalence of 6.6% at the baseline and of 3%, respectively, at 12 months following LSG, in contrast with the results of Dams-Machado [42] who indicated a decrease from 29% to 4.3%, and Shipton et al. [48], who also reported a decrease from 32.9% prior to LSG to 11.9% at 12 months postoperatively. Across our study period, the mean serum levels of iron increased significantly but remained within the normal range, in line with the low deficiency prevalence reported. Curiously, this variation was not influenced by sex or by the initial BMI, even though, compared to women, total body iron in men is three times higher. Several factors contribute to the development of LSG-mediated iron deficiency: hypochlorhydria, proton pump inhibitors intake, that further decreases stomach acid production, rapid gastric emptying, which reduces the contact between chyme and gastric juice, or the poor intake of animal protein due to food intolerance, which lowers the intake of heme iron, a readily absorbable form of iron, and promotes the decline in iron status [58].

Ferritin is the most sensitive and specific index of iron deficiency as it reflects a state of iron depletion [59]. Throughout the study duration, we observed no case of iron deficiency expressed in low ferritin serum levels. These findings are supported by Ruiz-Tovar et al. [25], who also reported no cases of ferritin deficiency neither at the baseline nor during the study follow-up period. Shipton et al. [60] found similar results, reporting no ferritin shortage at the baseline and a small number of de novo ferritin deficiency cases at 12 months after LSG (1.45%). Finally, a systematic review that analyzed 26 studies investigating the iron status post-LSG, out of which six used ferritin as a marker for iron deficiency, showed inconsistent results. As such, four studies reported no change in deficiency prevalence between the two time points, one revealed a decrease, and one showed an increase in iron deficiency at 12 months following LSG [43]. In agreement with these findings, we argue that iron status following LSG is markedly influenced by the baseline context and supplementation regimen.

Anemia, one of the most common long-term complications of all bariatric interventions [57], including LSG, has a reported prevalence that ranges between 0 and 30% prior to surgery and between 3.6 and 51% at 12 months postoperatively [43]. In our study, only 4.35% of patients presented anemia (based on low Hb levels) at the baseline, matching the results reported by Shipton et al. [60], with 4.6% cases of low Hb levels, and Silva Ferreira et al. [61], with 7.4% cases of anemia prior to LSG. In addition, we noted that neither Hb deficiency nor Hb mean values differed significantly between the two time points, in accordance with other reports [44,60]. The lack of change in anemia prevalence might be explained by the slight increase in iron levels that we observed across this study’s follow-up period.

All patients in our study presented 25(OH)D deficiency or insufficiency prior to LSG, which persisted by the end of the follow-up period as no significant change in 25(OH)D levels was depicted. Still, a significant increase (from 8.8 to 20.6 pmol/L) was observed from the first to the final time point in men but not in women. These data are comparable to previous research, showing that 25(OH)D hypovitaminosis is the most frequently reported deficiency in BS candidates with a prevalence up to 93% [17]. At the same time, in line with other studies [62], we also found that many patients with severe obesity exhibited 25(OH)D values below 10 pmol/mL before LSG, assumed to result most likely from inadequate sun exposure, improper storage, and bioavailability. Postoperatively, 25(OH)D status tends to improve according to previous LSG studies. However, we reported a different finding which might be explained by low adherence to postoperative supplementation and/or significantly low levels of 25(OH)D at the baseline. The current guidelines recommend supplementation with vitamin D to raise the serum concentration of 25(OH)D consistently over 30 ng/mL that is required to avoid alterations in calcium and PTH levels [18,31]. In our study, the calcium levels were within the normal range, exhibiting no significant change across the study period; however, we did notice significantly high calcium values in patients with an initial BMI ≥ 40 kg/m^2^. The PTH mean values also increased, although remaining within the normal range, at both the baseline and 12 months post-LSG, probably because of hypovitaminosis D that alters intestinal calcium absorption.

The present study has some limitations. One limitation is that we did not monitor the patients’ adherence to prescribed supplementation following LSG. Since these data were not considered, it is not clear whether the alterations in the micronutrient status were primarily induced by LSG itself or by low compliance to the postoperative supplementation regimen. Nor did we collect data about food intake or medication, which might also affect the micronutrient milieu. In addition, due to the small study group and missing data, caution is required in interpretation of these results, and future investigations on wider samples are needed to endorse the current data.

## 5. Conclusions

Our study provides evidence on the micronutrient profile of patients with severe obesity submitted to LSG and revealed significant changes at 12 months following surgery, except for vitamin B12. The 25(OH) D levels increased significantly in men but not in women during this study. Other micronutrient variations, however, were not influenced by sex or initial BMI. Within this study, no significant change in the frequency of MNDs was found, suggesting an overall normal micronutrient status in all patients. Moreover, the prevalence of anemia was low. Adequate supplementation and micronutrient monitoring are strongly recommended after LSG. Long-term adherence to supplementation is fundamental to prevent complications and optimize the outcomes of MBS.

## Figures and Tables

**Table 1:** Micronutrient-level deficiency or excess reference values.

Parameter	Deficiency	Excess
Iron	<50 μg/dL	-
Ferritin	<28 ng/mL (male) <5 ng/mL (female)	-
Folate	<2.7 ng/mL	-
Vitamin B_12_	<193 pg/mL	-
Calcium	<8.6 mg/dL	-
25(OH)D	<10 ng/mL	-
PTH		>64 pg/mL

**Table 2:** Baseline demographic and anthropometric characteristics of studied patients stratified by sex.

Variables	All Sample (*n* = 50)	Males (*n*_1_ = 16)	Females (*n*_2_ = 34)	*p*-Value
Age	46.5(10.9)	47.0 (11.1)	46.3(10.9)	0.83
BMI, kg/m^2^	43.1 (1.1)	43.5 (1.1)	42.9 (1.1)	0.75
WHR	0.9 (0.1)	1.1 (0.1)	0.9 (0.1)	<0.001 *
Comorbidities				
T2D, *n* (%)	12 (24)	5 (42)	7 (58)	0.49
Hypertension, *n* (%)	25 (50)	9 (36)	16 (64)	0.54
Dyslipidemia, *n* (%)	44 (88)	16 (36)	28 (64)	0.16
NAFLD, *n* (%)	31 (62)	10 (32)	21 (68)	0.96

Variables were summarized in terms of centrality and dispersion measure (arithmetic mean or geometric mean, standard deviation or geometric standard deviation), *n* = number of cases/sample sizes *n*_1_ = number of male subjects; *n*_2_ = number of female subjects; * significant result: *p* < 0.05. BMI: body mass index; WHR: waist–hip circumference ratio; T2D: type 2 diabetes; NAFLD: non-alcoholic fatty liver disease.

**Table 3:** Changes in anthropometric characteristics after LSG stratified by sex.

Outcome	Group	Baseline	6 MTH	*p*-Value ^1^	12 MTH	*p*-Value ^2^	*p*-Value ^3^	Adjusted *p*-Value (Male vs. Female)		
		LS-Means [95% CI]	LS-Means [95% CI]		LSMs [95% CI]			Baseline	6 MTH	12 MTH
Weight (kg) ^a^	Male	139.8 [130.3, 149.9] ^#^	98.5 [91.8, 106.7] ^#^	<0.001 *	91.8 [85.6, 99.5] ^#^	0.03 *	<0.001	<0.001 *	0.004 *	<0.001 *
	Female	114.4 [108.9, 120.3] ^#^	83.1 [79.1, 87.4] ^#^	<0.001 *	73.7 [70.1, 78.3] ^#^	<0.001 *	<0.001 *			
BMI, kg/m^2 a^	Male	43.8 [41.4, 46.2]	31.2 [29.6, 32.9]	<0.001 *	28.9 [26.4, 31.4]	0.13	<0.001 *	>0.99	1.00	0.98
	Female	43.3 [41.6, 44.9]	31.0 [28.5, 33.4]	<0.001 *	27.8 [26.1, 29.5]	<0.001 *	<0.001 *			
WHR ^b^	Male	1.06 [1.02, 1.10]	1.03 [0.99, 1.07]	0.58	1.01 [0.96, 1.06]	0.94	0.19	<0.001 *	<0.001 *	0.002 *
	Female	0.93 [0.91, 0.96]	0.91 [0.89, 0.94]	0.75	0.90 [0.87, 0.93]	0.94	0.22			

*n* = number of patients; *n*_1_ = number of patients in the first group; *n*_2_ = number of patients in the second group; 6 MTH = at 6 months and 12 MTH = at 12 months after LSG; ^1^ adjusted *p*-values for change from baseline to 6 MTH; ^2^ adjusted *p*-values for change from 6 MTH to 12 MTH; ^3^ adjusted *p*-values for change from baseline to 12 MTH; * significant result: *p* < 0.05; ^a^ baseline: *n* = 50 (*n*_1_ = 16, *n*_2_ = 34), 6 MTH: *n* = 50 (*n*_1_ = 16, *n*_2_ = 34), 12 MTH: *n* = 45 (*n*_1_ = 14, *n*_2_ = 31); ^b^ baseline: *n* = 50 (*n*_1_ = 16, *n*_2_ = 34), 6 MTH: *n* = 49 (*n*_1_ = 15, *n*_2_ = 34), 12 MTH: *n* = 40 (*n*_1_ = 10, *n*_2_ = 30); 95% CI = 95% confidence intervals were estimated from LMMs or GLMMs adjusted for age and having patients as random effect; ^#^ estimated least-square means (LS-means) for log-normal data are given on the response scale (after we exponentiated the results obtained for log scale); *p*-values at each time point were estimated using Tukey’s HSD correction.

**Table 5:** Frequencies (absolute and relative) of MNDs over 12 months after LSG.

	Preoperative Deficiency *n* (%)	Postoperative Deficiency ^1^ *n* (%)	*p*-Value
Vitamin B_12_ (pg/mL) ^a^	8 (16.7)	6 (12.5)	0.75
Folate (ng/mL) ^b^	3 (6.7)	5 (11.1)	0.68
Iron (μg/dL) ^c^	1 (2.3)	2 (4.6)	1.00
Ferritin (ng/mL)	0 (0.0)	0 (0.0)	Na
25(OH)D (ng/mL) ^d^	15 (100.0)	15 (100.0)	Na
Calcium (mg/dL) ^e^	2 (4.7)	1 (2.3)	1.00

*n* = number of cases; ^1^ measured at 12 months after LSG; ^a^ complete case data for baseline and 12 months after LSG *n* = 48; ^b^ complete case data for baseline and 12 months after LSG *n* = 45; ^c^ complete case data for baseline and 12 months after LSG *n* = 44; ^d^ complete case data for baseline and 12 months after LSG *n* = 15; ^e^ complete case data for baseline and 12 months after LSG *n* = 43; Na = not available; *p*-values were obtained from the McNemar test.

## Data Availability

The original contributions presented in this study are included in the article and Supplementary Material. Further inquiries can be directed to the corresponding authors.

## Supplementary Materials

The following supporting information can be downloaded at: https://www.mdpi.com/article/10.3390/nu16244386/s1, Table S1: Changes in hematinic and hematological values after LSG stratified by sex and initial BMI level; Table S2: Between-group (sex and BMI) pre–post LSG difference concerning studied micronutrients obtained using linear mixed effect models.
