# Supplementary material for: Micronutrient Status in Patients with Severe Obesity Before and After Laparoscopic Sleeve Gastrectomy
Source: Nutrients. 2024 Dec 20;16(24):4386. doi: 10.3390/nu16244386 (PMC11678899; doi:10.3390/nu16244386)
Supplement: Supplementary file 1 [file nutrients-16-04386-s001.zip › nutrients-3322989-supplementary.pdf]

Supplemental Table

Table S1. Changes in hematinic and hematological values after LSG stratified by sex and initial BMI level

Outcome	Group	Baseline LS-means [95% CI]	6 MTH LS-means [95% CI]	p-value (1	12 MTH LS-means [95% CI]	p-value (2	p-value (3	Adjusted p-values (male vs. female and respectively BMI ≥ 40 vs. BMI <40 kg/m²): Baseline	Adjusted p-values: 6 MTH	Adjusted p-values: 12 MTH
Vitamin B_{12} (pg/mL) (a	Male	324.1 [210.9, 498.0] #	373.3 [255.5, 545.5] #	0.96	377.8 [251.8, 566.9]#	1.00	0.95	0.97	0.96	0.74
	Female	365.1 [278.4, 478.8] #	331.5 [246.7, 445.3] #	0.96	309.7 [224.4, 427.5]#	>0.99	0.80			
	BMI≥40 kg/m²	347.8 [264.9, 456.5] #	352.4 [270.9, 458.3] #	>0.99	371.5 [288.0, 479.2]#	>0.99	0.99	>0.99	>0.99	0.93
	BMI 35-40 kg/m²	340.2 [221.7, 522.1] #	351.1 [229.8, 536.5] #	1.00	315.0 [190.7, 520.4]#	>0.99	>0.99			
Folate (ng/mL) (b	Male	5.9 [3.4, 10.1] #	9.0 [5.8, 14.1] #	0.07	5.4 [3.0, 9.7] #	0.04*	>0.99	1.00	>0.99	0.98
	Female	5.8 [3.8, 8.7] #	8.4 [6.1, 11.7] #	0.04*	6.2 [4.2, 9.4] #	0.17	>0.99			
	BMI≥40 kg/m²	6.0 [4.1, 8.8] #	8.0 [5.8, 11.0] #	0.17	6.9 [4.7, 10.0] #	0.77	0.91	>0.99	0.91	0.67
	BMI 35-40 kg/m²	5.7 [3.2, 10.1] #	9.5 [3.2, 14.9] #	0.02*	4.9 [2.6, 9.3] #	0.007*	0.99			
Iron (μg/dL) (c	Male	98.5 [82.0, 115.0]	104.7 [88.2, 121.1]	0.99	129.2 [112.0, 146.3]	0.14	0.03*	0.32	0.81	0.03*
	Female	78.6 [66.8, 90.4]	92.5 [81.0, 104.0]	0.34	98.5 [86.7, 110.2]	0.96	0.05			
	BMI≥40 kg/m²	90.2 [79.3, 101.0]	99.2 [88.3, 110.0]	0.73	109.3 [97.7, 121.0]	0.67	0.06	>0.99	1.00	0.95
	BMI 35-40 kg/m²	86.9 [69.2, 105.0]	98.0 [80.7, 115.0]	0.90	118.3 [101.0, 136.0]	0.03*	0.35			
Ferritin (ng/mL) (d	Male	267.0 [210.0, 324.0]	219.0 [171.6, 267.0]	0.52	196.0 [138.6, 253.0]	0.96	0.32	0.003*	<0.001*	0.05
	Female	136.0 [91.1, 181.0]	102.0 [68.8, 134.0]	0.62	103.0 [69.1, 138.0]	1.00	0.70			
	BMI≥40 kg/m²	197.0 [158.3, 236.0]	190.0 [158.1, 222.0]	>0.99	167.0 [131.1, 202.0]	0.77	0.73	>0.99	0.32	0.88
	BMI 35-40 kg/m²	206.0 [140.3, 272.0]	131.0 [81.7, 180.0]	0.19	133.0 [77.2, 188.0]	1.00	0.32			
Hb (g/L) (e	Male	15.4 [14.8, 16.0]	14.7 [14.1, 15.3]	0.09	14.5 [13.9, 15.1]	0.98	0.02*	<0.001*	0.012*	<0.001*
	Female	13.6 [13.2, 14.0]	13.3 [12.9, 13.7]	0.69	13.0 [12.6, 13.4]	0.42	0.02*			
	BMI≥40 kg/m²	14.8 [14.4, 15.2]	14.1 [13.8, 14.5]	0.002*	14.0 [13.6, 14.4]	0.88	< 0.001*	>0.99	0.98	0.88
	BMI 35-40 kg/m²	14.1 [13.5, 14.8]	13.9 [13.3, 14.5]	0.93	13.6 [12.9, 14.2]	0.82	0.24			
Hct (%) (e	Male	45.0 [43.4, 46.6]	43.7 [42.1, 45.4]	0.52	42.9 [41.3, 44.6]	0.87	0.06	0.001*	<0.001*	<0.001*
	Female	40.5 [39.3, 41.6]	39.7 [38.5, 40.8]	0.64	38.8 [37.7, 40.0]	0.53	0.02*			

	BMI≥40 kg/m²	43.7 [42.7, 44.8]	41.9 [40.8, 43.0]	0.002*	41.4 [40.3, 42.6]	0.95	<0.001*	0.31	>0.99	0.86
	BMI 35-40 kg/m²	41.7 [40.0, 43.4]	41.6 [39.8, 43.3]	1.00	40.3 [38.6, 42.0]	0.56	0.44			
MCV (fL) (e	Male	89.3 [87.9, 90.7]	90.9 [88.9, 93.0]	0.24	90.0 [88.0, 92.1]	0.83	0.93	1.00	1.00	>0.99
	Female	89.3 [87.9, 90.7]	90.8 [89.3, 92.2]	0.06	89.8 [88.3, 91.2]	0.39	0.95			
	BMI≥40 kg/m²	89.7 [88.3, 91.0]	90.8 [89.4, 92.1]	0.22	90.3 [89.0, 91.7]	0.95	0.78	0.99	1.00	0.98
	BMI 35-40 kg/m²	88.9 [86.8, 91.1]	90.9 [88.8, 93.1]	0.11	89.5 [87.3, 91.6]	0.40	0.99			
MCH (pg) (e	Male	30.6 [29.7, 31.4]	30.8 [30.0, 31.7]	0.99	30.6 [29.7, 31.5]	>0.99	1.00	0.85	1.00	0.88
	Female	30.0 [29.4, 30.6]	30.9 [30.3, 31.5]	0.0498*	30.1 [29.4, 30.7]	0.11	>0.99			
	BMI≥40 kg/m²	30.4 [29.8, 31.0]	30.9 [30.3, 31.5]	0.50	30.4 [29.8, 31.0]	0.67	>0.99	>0.99	1.00	>0.99
	BMI 35-40 kg/m²	30.1 [29.2, 31.1]	30.8 [29.9, 31.7]	0.74	30.2 [29.3, 31.2]	0.85	1.00			

n=number of patients; n_1=number of patients in the first subgroup; n_2=number of patients in the second subgroup; 6 MTH = at six months and 12 MTH = at twelve months after LSG; (1 adjusted-p values for change from baseline to 6 MTH; (2 adjusted-p values for change from 6 MTH to 12 MTH; (3 adjusted-p values for change from baseline to 12 MTH;*significant result: p<0.05; (a T0: n=50 (Sex: n_1 =16, n_2 = 34; BMI: n_1 =36, n_2 = 14), T6: n=50 (Sex: n_1 =16, n_2 = 34; BMI: n_1 =36, n_2 = 14),, T12: n=48 (Sex: n_1 =14, n_2 = 34; BMI: n_1 =35, n_2 = 13),; (b T0: n=50 (Sex: n_1 =16, n_2 = 34; BMI: n_1 =36, n_2 = 14), T6: n=50 (Sex: n_1 =13, n_2 = 32; BMI: n_1 =32, n_2 = 13), T12: n=45 (Sex: n_1 =13, n_2 = 32; BMI: n_1 =35, n_2 = 13), (c T0: n=49 (Sex: n_1 =16, n_2 = 33; BMI: n_1 =36, n_2 = 13), T6: n=50 (Sex: n_1 =16, n_2 = 34; BMI: n_1 =36, n_2 = 14), T12: n=45 (Sex: n_1 =14, n_2 = 31; BMI: n_1 =31, n_2 = 14); (d T0: n=25 (Sex: n_1 =10, n_2 = 15; BMI: n_1 =19, n_2 = 6), T6: n=49 (Sex: n_1 =15, n_2 = 34; BMI: n_1 =35, n_2 = 14), T12: n=38 (Sex: n_1 =9, n_2 = 29; BMI: n_1 =27, n_2 = 11); (e T0: n=50 (Sex: n_1 =16, n_2 = 34; BMI: n_1 =36, n_2 = 14), T6: n=50 (Sex: n_1 =16, n_2 = 34; BMI: n_1 =36, n_2 = 14), T12: n=46 (Sex: n_1 =15, n_2 = 31; BMI: n_1 =32, n_2 = 14); 95% CI=95% Confidence Interval, Marginal means were estimated from LMM or GLMM adjusted for age and having patients as random effect; # estimated least square means (LS-means) for log-normal data are given on the response scale (after we exponentiated the results obtained for log scale); p-values at each time point were estimated using Tukey's HSD correction.

Table S2. Between-group (sex and BMI) pre–post LSG difference concerning studied micronutrients obtained using linear mixed effect models.

	**Estimate (β) Time[1] * Sex[3]**	**SE**	**p-interaction**	**Estimate (β) Time[2] * Sex[3]**	**SE**	**p-interaction**	**Estimate (β) Time[1]*BMI[4]**	**SE**	**p-interaction**	**Estimate (β) Time[2]*BMI[4]**	**SE**	**p-interaction**
Anthropometric characteristics												
%EWL	Na	Na	Na	-9.34	2.96	0.003*	Na	Na	Na	Na	Na	Na
%EBMIL	Na	Na	Na	-9.27	2.94	0.003*	Na	Na	Na	Na	Na	Na
%TWL	Na	Na	Na	-3.61	1.33	0.009*	Na	Na	Na	Na	Na	Na
Hematinic and hematological characteristics												
Vitamin B_{12} (pg/mL)	0.25	0.20	0.22	0.32	0.20	0.12	0.011	0.21	0.96	0.16	0.22	0.49
Folate (ng/mL)	0.06	0.20	0.78	-0.16	0.23	0.50	-0.23	0.20	0.26	0.27	0.25	0.28
Iron (µg/dL)	-7.69	11.27	0.50	10.83	11.71	0.36	-2.06	11.87	0.86	-12.29	12.04	0.31
Ferritin (ng/mL)	-13.46	32.78	0.68	-38.53	38.58	0.32	68.11	36.58	0.07	43.40	39.44	0.28
Hb (g/L)	-0.41	0.27	0.16	-0.26	0.29	0.39	-0.41	0.30	0.17	-0.28	0.30	0.36
Hct (%)	-0.47	0.83	0.57	-0.42	0.85	0.62	-1.74	0.86	0.046*	-0.92	0.87	0.29
MCH (pg)	-0.68	0.54	0.21	-0.02	0.55	0.97	-0.13	0.56	0.82	-0.03	0.57	0.95
MCV (fl)	0.15	0.84	0.86	0.26	0.87	0.77	-0.88	0.87	0.32	0.14	0.89	0.87
Vitamin D-calcium status and PTH												
25(OH)D (ng/mL)	0.25	0.21	0.24	0.78	0.21	<0.001*	0.05	0.25	0.84	-0.17	0.25	0.49
PTH (pg/mL)	-3.32	4.92	0.50	-1.71	5.34	0.75	-5.79	5.19	0.27	-4.85	5.40	0.37
Calcium (mg/dL)	0.03	0.15	0.85	0.23	0.16	0.15	0.11	0.15	0.48	0.39	0.16	0.02*

[1] T6 vs. T0 ; [2] T12 vs. T0 ; [3] Sex : male versus female ; [4] BMI : BMI≥40 vs.35-40 kg/m^2; Na= not applicable; β = estimated coefficient of Linear Mixed Model or Generalized Linear Mixed Models with time (T0, T6, T12) and group (Sex and BMI) as fixed effects and patient as random effect; the estimated coefficients were used in determination of age-adjusted estimated least-square means (LS-means). All linear mixed models were adjusted for age; SE= standard error of the estimate coefficient; * significant result: p- value < 0.05 (p-value of the null hypothesis of β=0);
